# Mapping Brain Spatial Metabolic Atlas Reveals Anesthesia-Induced Region-Specific Alterations

**DOI:** 10.1007/s12264-026-01675-6

**Published:** 2026-07-18

**Authors:** Wei Feng, Lijuan Pang, Chun-Jie Liu, Jiuming He, Lisi Wang, Zhenyu Xu, Yinglong Zeng, Zengqiang Yuan, Chao Zhang

**Affiliations:** 1 Zhanjiang Institute of Clinical Medicine, Central People’s Hospital of Zhanjiang, Zhanjiang, 524045 China; 2 Zhanjiang Central Hospital, Guangdong Medical University, Zhanjiang, 524045 China; 3 Center for Artificial Intelligence Biology, Hubei Bioinformatics and Molecular Imaging Key Laboratory, Key Laboratory of Molecular Biophysics of the Ministry of Education, College of Life Science and Technology, Huazhong University of Science and Technology, Wuhan, 430074 China; 4 State Key Laboratory of Bioactive Substance and Function of Natural Medicines, Institute of Materia Medica, Chinese Academy of Medical Sciences and Peking Union Medical College, Beijing, 100050 China; 5 Shanghai Luming Biological Technology Co., Ltd., Shanghai, 201114 China; 6 Department of Pathology, Zhanjiang Central Hospital, Guangdong Medical University, Zhanjiang, 524000 China; 7 Beijing Institute of Basic Medical Sciences, Beijing, 100850 China; 8 School of Basic Medical Sciences, Hengyang Medical School, University of South China, Hengyang, 421001 China

**Keywords:** Brain, Anesthesia, Metabolism, Mass spectrometry, Imaging

## Abstract

The brain supports consciousness through regional heterogeneity. However, the spatial complexity of its metabolome under anesthesia remains unclear. In this study, we established a spatial metabolomic atlas of the mouse brain under wakefulness, inhalation anesthesia, and injectable anesthesia. In total, 727 metabolites were identified and spatially mapped across 11 representative brain regions. We found that anesthesia significantly altered the brain metabolic landscape in a spatially dependent manner, particularly affecting lipid metabolism, amino acid pathways, and neurotransmitter-related metabolites. Furthermore, we monitored the cortical microvascular blood flow and blood oxygen levels during anesthesia. We observed that different anesthesia modalities induced distinct changes in cortical arteriovenous microvascular hemodynamics, suggesting a potential association between metabolic alterations and cerebrovascular regulation. We developed an open-access online database, the mouse brain spatial metabolome atlas (https://mbsma.liulab.cloud/#/dashboard), to facilitate access to and analysis of the dataset. Our study reveals anesthesia-induced spatial metabolic reprogramming in the brain, providing insights into neural metabolism and cerebrovascular dynamics, with implications for perioperative brain protection and safer anesthesia strategies.

**Supplementary Information:**

The online version contains supplementary material available at https://doi.org/10.1007/s12264-026-01675-6.

## Introduction

The brain is the most structurally and functionally complex organ in mammals and is a source of consciousness [1–4]. Therefore, understanding brain function is one of the most important topics in the scientific community. There is unique heterogeneity in the brain, characterized by intricate functional connectivity and marked diversity among cell types in different brain regions [5–8]. The communication network among brain regions is the basis of brain function, and the interactions between cells, intrinsic genetic programs, and signaling molecules in the brain exhibit complex spatial diversity. Spatial omics is a novel strategy for revealing the complex structural and functional heterogeneity of the brain, transforming our understanding of the brain from a regional perspective to a systemic perspective [9–11]. Among them, spatial transcriptomics and proteomics have been widely used to map gene transcripts and proteins in the brain [12–17].

Metabolites can reflect biochemical processes at the systems level (e.g., cells, tissues, or organs), which cannot be simply predicted using genomic or proteomic markers [18–23]. To date, the brain metabolome has not been sufficiently investigated. Recently, spatial metabolic networks of the rat brain were mapped using high-throughput ambient airflow-assisted desorption electrospray ionization mass spectrometry imaging (MSI) [24]. That study obtained 298 and 372 monoisotopic ion images in the positive and negative ion modes, respectively, and mostly focused on a few small molecules in the rat brain (mass-to-charge ratio of 50–500 Da). However, genetic differences between rats and mice are substantial [25] and can manifest as functional and chemical differences between their brains [26–28]. In addition, most neuroscience research has been conducted in mice because they provide access to a much larger genetic toolbox. Therefore, more attention should be paid to the spatial metabolomic atlas of the mouse brain.

A previous metabolome atlas of the mouse brain across different ages provided data on 1,547 metabolites from 10 typical brain regions [29]. However, in that study, manual surgical extraction of brain anatomical regions during sample preparation was time-consuming. Additionally, the brain, which has high metabolic activity, exhibits a rapid rate of postmortem metabolomic transformation, which may lead to severe degradation of small molecule metabolites during sample dissection [30, 31]. Furthermore, to avoid contamination at the borders between brain regions, certain parts of the brain were excluded, and the spatial distribution of metabolites within these discarded regions was not considered.

Anesthesia has profound and state-dependent effects on metabolism, making it a critical factor to consider in brain metabolomics research. Different anesthetics have been reported to lead to different cerebral hemodynamic and neural activity changes [32, 33], suggesting differences in brain metabolic activity under various anesthetics. A previous study showed that the anesthetized and awake mouse prefrontal cortices differ in their neurochemistry [34]. However, this partial analysis cannot comprehensively reveal the metabolic variations among different brain regions induced by anesthetics, let alone compare the brain region-specific metabolic differences caused by various anesthetics. Thus, mapping the spatial metabolome atlas of the mouse brain is crucial for understanding the differences and similarities in wakefulness- and anesthesia-related brain metabolism. This endeavor contributes to a deeper understanding of how anesthesia reprograms regional brain metabolism and aids in elucidating the metabolic pathways through which anesthesia modulates neuronal function. This provides valuable information for the development of safer anesthetic strategies and holds significant clinical importance.

In this study, we employed a desorption electrospray ionization-based MSI technique to map the wakefulness- and anesthesia-related spatial metabolome atlas of the mouse brain. A total of 727 metabolites were identified and annotated by manual curation of data integrated from public databases, in-house repositories, and liquid chromatography-tandem mass spectrometry (LC-MS/MS) analysis. A comparative analysis of wakefulness and two anesthetic modalities often employed in animal investigations revealed anesthesia-induced alterations in the spatial distribution of brain metabolites. A noninvasive awake animal imaging approach was employed to monitor the hemodynamic responses of cortical arteriovenous microvessels from wakefulness to anesthesia. Additionally, a free online database was established to facilitate researchers’ access to brain spatial metabolome data (https://mbsma.liulab.cloud/#/dashboard).

## Materials and Methods

### Animals

The experimental procedures were performed with the approval of the Guangdong Medical University Animal Ethics Committee (Approval number: GDY2002085) and were conducted in compliance with the animal experiment guidelines of the Experimental Animal Management Ordinance of Guangdong Province, P. R. China, General Requirements for Laboratory Animal Experiments of P. R. China (GB/T 35823-2018), and the ARRIVE guidelines. This study was approved by the Institutional Animal Ethics Committee of the Central People's Hospital of Zhanjiang. Mice, purchased from Guangdong Zhiyuan Biomedical Technology Co., Ltd., were fed under specified pathogen-free conditions with a 12-hour light/dark cycle. Spatial metabolomic analysis was performed on brain samples from littermates of 8‑week‑old male C57BL/6 mice (*n* = 3 per group), while 8‑week‑old male C57BL/6 mice weighing 18–22 g were used for *in vivo* cerebral vascular imaging, electroencephalography, and heart rate monitoring (*n* = 5–6 per group).

### Anesthesia

Two types of mouse anesthetics were used in this study. For inhalation anesthesia, 2% (flow rate, 0.5 mL/min) isoflurane (RWD Life Science Co., Ltd., Shenzhen, China) was continuously administered. For the injectable anesthetic, the stock solution was prepared by dissolving 10 g of 2,2,2-tribromoethanol (Avertin) (T48402, Sigma-Aldrich, St. Louis, MO, USA) in 10 mL of tertiary amyl alcohol (240486, Sigma-Aldrich, St. Louis, MO, USA). Before use, it was diluted with saline (1:40) and maintained at 4°C away from light. The anesthetic was administered intraperitoneally (0.02 mL/g) immediately before use. The degree of anesthesia was assessed based on the toe-pinch reflex.

In addition to the toe-pinch reflex, electroencephalography (EEG) and electrocardiography (ECG) were performed using a Medusa system (Yige Biotechnology Co., Ltd., Nanjing, China). EEG signals and heart rates were automatically analyzed using SleepScore and Heartjump software (Yige Biotechnology Co., Ltd., Nanjing, China).

Tissue collection was performed at standardized time points after anesthesia induction. In the anesthetic group, mice were maintained under a stable state of anesthesia before decapitation (typically 20 min after inhalation or intraperitoneal administration).

### Sample Preparation for Liquid Chromatography–Tandem Mass Spectrometry (LC-MS/MS)

The brain samples stored at −80°C were thawed and ground at room temperature. Twenty microliters of L-2-chlorophenylalanine (0.06 mg/mL) dissolved in methanol was used as an internal standard, along with 30 mg of the sample in a 1.5 mL Eppendorf tube. The samples were then extracted using ultrasonic technology for 10 min in an ice-water bath and kept at −20°C for 30 min. The extract was centrifuged for 10 min (4°C, 13,000 r/min). A freezing concentration centrifugal drier was used to dry 300 μL of supernatant in a glass vial. Each sample was redissolved in a 300 μL mixture of methanol and water (vol/vol = 1:4) before being vortexed for 30 s; it was then ultrasonically extracted for 3 min in an ice-water bath and stored at −20°C for 2 h, after which it was centrifuged for 10 min (4°C, 13,000 r/min). Using crystal syringes, supernatants (150 μL) from each tube were collected, filtered through microfilters (0.22 μm), and then transferred to LC vials. The vials were stored at −80°C until LC-MS analysis. QC samples were prepared by mixing aliquots of all samples to form a pooled sample.

### LC-MS/MS Analysis and Data Preprocessing

The ACQUITY UPLC I-Class Plus (Waters Corporation, Milford, MA, USA), equipped with a Q-Exactive mass spectrometer and a heated electrospray ionization (ESI) source (Thermo Fisher Scientific, Waltham, MA, USA), was used to analyze the metabolic profile in both ESI positive and ESI negative ion modes, and an ACQUITY UPLC HSS T3 column (2.1 × 100 mm, 1.8 μm) was employed. The binary gradient elution system consisted of (A) water (containing 0.1% formic acid, vol/vol) and (B) acetonitrile (containing 0.1% formic acid, vol/vol), and separation was accomplished using the following gradient program: 0.01 min, 5% B; 2 min, 5% B; 4 min, 30% B; 8 min, 50% B; 10 min, 80% B; 14 min, 100% B; 15 min, 100% B; 15.1 min, 5% B; and 16 min, 5% B. The column temperature was 45°C, and the flow rate was 0.35 mL/min. All the samples were kept at 4°C during the analysis, and the injection volume was 2 μL.

The mass range was *m/z* 100–1200. The full-scan resolution was set to 70,000, and the higher-energy collisional dissociation (HCD) MS/MS scan resolution was set to 17,500. The mass spectrometer was operated as follows: spray voltage, 3800 V (positive ion) and 3000 V (negative ion); sheath gas flow rate, 40 arbitrary units (positive ion) and 35 arbitrary units (negative ion); auxiliary gas flow rate, 10 arbitrary units (positive ion) and 8 arbitrary units (negative ion); and capillary temperature, 320°C (positive ion) and 320°C (negative ion).

The raw data were processed using Progenesis QI V2.3 (Nonlinear Dynamics, Newcastle, UK) for baseline filtering, peak identification, integration, retention time correction, peak alignment, and normalization. The main parameters were set as a 5 ppm precursor tolerance, a 10 ppm product tolerance, and a 5% product ion threshold. Compounds were identified based on precise m/z, secondary fragments, and isotopic distributions using the Human Metabolome Database (HMDB), LipidMaps (V2.3), METLIN, and an in-house SmetDB database (Luming Bio, Shanghai, China). The extracted data were further processed to remove peaks in any group that exceeded 50% missing values (ionic intensity of 0), replace 0 with half of the minimum value, and screen compounds according to the qualitative results. A compound was declared erroneous and eliminated if the resulting score was less than 36 (out of 60). Finally, the valid positive and negative ion data were integrated into a data matrix containing all information extracted from the raw data.

### Chemicals and Reagents for Mass Spectrometry Imaging (MSI)

MS-grade acetonitrile was purchased from Thermo Fisher (Thermo Fisher, USA). Purified water was obtained from Watson (Hong Kong, China). HPLC-grade formic acid was purchased from Merck (Kenilworth, NJ, USA). Tissue-freezing medium was obtained from Leica Microsystems (Germany). Eosin Y solution (0.5% aqueous solution) and hematoxylin were purchased from Sigma-Aldrich (St. Louis, MO, USA). Hematoxylin and bluing buffer (Dako, Shanghai Qizhong Information Technology Co., LTD., China) were used.

### Sample Preparation for MSI

Mouse brains were harvested following cervical dislocation in the wake group and following the induction of deep anesthesia in the anesthetic group (*n* = 3 per group). To minimize the impact of euthanasia on brain metabolism, the total time from cervical dislocation to tissue harvesting was controlled to within 90 s for all experimental groups. Both the wake and anesthetic groups were strictly maintained with a consistent post‑euthanasia interval (i.e., the time from euthanasia to tissue freezing), which ranged from 60 to 90 s. Brain samples were washed with cold saline and then kept at −80°C before sectioning. The samples were sectioned into 10 μm successive sagittal slices using a cryostat microtome (Leica CM 1950, Leica Microsystems, Germany) and then placed on positively charged desorption plates (Thermo Scientific, USA) after thawing. Sections were stored at −80°C until further analysis. Before MSI analysis, slices were desiccated at −20°C for 1 h and subsequently at room temperature for 2 h. A nearby slice was stained with hematoxylin and eosin (H&E) in the interim.

### Data Acquisition for MSI

An AFADESI-MSI platform (Beijing Victor Technology Co., Ltd., Beijing, China) and a Q-Orbitrap mass spectrometer (Q Exactive, Thermo Scientific, USA) were used in this study. The solvent compositions used for this experiment were acetonitrile/water (vol/vol = 8:2) in the negative mode and acetonitrile/water (vol/vol = 8:2; 0.1% formic acid) in the positive mode. The solvent flow rate was 5 μL/min, the transporting gas rate was 45 L/min, and the spray voltage was set to 7 kV. The distance between the sprayer and the sample surface was 0.7 mm. The distance between the sprayer and the ion-transporting tube was 3 mm. The MS resolution was set at 70,000, the mass range was 70–1000 Da, the automated gain control (AGC) target was 2 × 10^6^, and the maximum injection duration was set to 200 ms. The S-lens voltage was 55 V, and the capillary temperature was 350°C. The MSI experiment was performed at a constant rate of 0.2 mm/s to continuously scan the surface of the brain section in the x direction and with a 100 μm vertical step in the y direction.

### Data Preprocessing and Analysis for MSI

The raw data were then converted to .imzML format using imzMLConverter [35] and imported into MSiReader (an open-source interface for viewing and analyzing high-resolution MSI data on the MATLAB platform). Ion image reconstruction was performed after background subtraction using the Cardinal software package [36]. Total ion count normalization for each pixel was used to normalize all MS images [37]. High-spatial-resolution H&E staining allowed for the extraction of region-specific MS characteristics. A supervised statistical analytical technique called orthogonal partial least squares discrimination analysis (OPLS-DA) was used to identify discriminating endogenous chemicals in various microregions of the brain. The variable importance of projection (VIP) values from the OPLS-DA model were used to rank the total contribution of each variable to group discrimination. A VIP value greater than 1 indicates that the variable has a considerable impact on the classification of sample categories with regard to the first two principal components of the OPLS-DA model. Further analysis was performed to determine whether the differences in metabolites between the groups were statistically significant, using a two-tailed Student's *t*-test. VIP scores greater than 1.0 and *P*-values less than 0.05 were used to select differential metabolites. In addition, to minimize model overfitting, a seven-fold cross-validation (7-fold CV) strategy and response permutation testing (RPT, *n* = 200) were performed to evaluate the robustness and predictive reliability of the OPLS-DA models. Permutation testing was conducted by maintaining the X matrix constant while randomly permuting the class labels in the Y matrix (e.g., 0/1) 200 times. For each permutation, a new OPLS-DA model was constructed to generate the corresponding R^2^ and Q^2^ values for the randomized models. Linear regression was performed between the R^2^Y and Q^2^Y values of the original model and those derived from the permuted models. The intercepts of the resulting regression lines on the y-axis (R^2^ and Q^2^) were used to evaluate whether the model was overfitted (Supplementary Data 1 shows the model parameters for the multivariate statistical analysis). The ions detected by AFADESI-MSI were annotated using the pySM pipeline [38], with reference to HMDB, LipidMaps (V2.3), METLIN, and an in-house SmetDB database.

Batch correction was performed on all samples using the Harmony method [39]. For comparative analysis, we employed an intragroup resampling method to calculate the mean. For each group of pixels, 10% were randomly selected to calculate the mean, and the process was repeated 100 times within each group. For each brain region (11 brain regions) in each group (three samples), 10% of the pixels in the same brain region were selected each time to obtain the mean value. Principal component analysis (PCA) of each brain region was performed using the normalized sample mean (combined with positive and negative ion modes), and PCA was performed at 99 points (3 × 3 × 11). Enrichment analysis of the metabolic pathways was performed using the Kyoto Encyclopedia of Genes and Genomes (KEGG). Each time, 10% of the pixels were selected from all the pixels in the same brain region to obtain the mean value. The correlation analysis was performed 10 times (330 mean data points [3 × 11 × 10] in a single group), and the Pearson correlation method was used for correlation analysis of brain regions in the same group. For correlation analysis among different groups in the same brain region, nine samples were taken; all pixel points in each sample were selected to obtain the average value of 10% of the pixel points each time, and this process was repeated 10 times (9 × 10 mean data), after which the Pearson correlation analysis method was performed.

### Establishment of the Long-Term Clearing Cranial Window (LCCW)

The cranial window, named the LCCW, was developed to maintain the native brain microenvironment and enable high-resolution cortical vasculature imaging. The detailed establishment procedure was reported in our previous work [40]. *In vivo* skull optical clearing agents, S1 and S2, were successively applied to make the skull transparent. S1 is a saturated solution consisting of urea (Sinopharm, China) and 75% ethanol (Sinopharm, China) with a volume-to-mass ratio of approximately 10:3. S2 was prepared by mixing dodecylbenzenesulfonic acid (DDBSA, Aladdin, China) with a 0.7 mol/L NaOH solution, and the volume-to-mass ratio was approximately 24:5. The mouse head hair and scalp were removed, and a skull holder was glued onto the dried skull. Then, S1 was applied, and the skull was gently rubbed with a small piece of cotton until the cortical vessels became visible. Next, S1 was removed, and S2 was applied for 5 min to make the skull transparent.

The transparency of the skull was maintained using a skull-clearing maintenance reagent. It consisted of a primer gel (Superior Mixing Clear Gel, Ryuji and May, China), a joint gel (K-303, Kafuter, China), and a sealing gel (Plated Crystal Antifouling Sealing Gel, Rainey, China). The cleared skull was first dried; then, the primer gel and joint gel were added and mixed uniformly, and a 365 nm, 5 W LED light was employed to solidify the mixture at a distance of 30 cm. Subsequently, a thin layer of sealing gel was applied and solidified under the same light. Approximately 2 μL of priming gel, 20 μL of joint gel, and 2 μL of sealing gel were needed to establish an LCCW with a 5 mm diameter.

### Monitoring Cerebrovascular Hemodynamic Changes During Anesthesia

We integrated hyperspectral imaging (HSI) and laser speckle contrast imaging (LSCI) to monitor vascular blood flow and blood oxygen levels simultaneously. This dual-mode imaging system mainly consists of two charge-coupled device (CCD) cameras (Panda4.2BI, PCO Company, Germany), a liquid crystal tunable filter (LCTF, CRi Varispec VIS, Perkin Elmer, USA), a stereomicroscope (SMZ1270, Nikon, Japan), a ring-shaped LED light with a polarizer, a filter (633 ± 5 nm), and a He-Ne laser beam (λ = 632.8 nm, 3 mW) expanded by a collimating lens used to illuminate the areas of interest. The LCTF has a bandwidth of 7 nm. Standard hyperspectral images were captured using a certified reflectance standard (SRS-99-020, LabSphere, USA). All hyperspectral images were collected between 500 and 620 nm with a step size of 10 nm. The algorithms used to calculate blood flow from the raw speckle dataset and blood oxygen saturation from the hyperspectral dataset were based on previous studies [41, 42]. The LCCW and dual-mode imaging systems were combined to track dynamic changes in blood oxygen and cerebrovascular blood flow during the administration of various anesthetics.

The LCCW was established three days before monitoring cerebrovascular blood flow and oxygen dynamics under different anesthesia treatments. For inhalation anesthesia, cerebrovascular hemodynamic changes were first recorded for 5 min while the mice were awake, then under inhalation anesthesia for 40 min, followed by another 55 min of observation after isoflurane was terminated. For injection anesthesia, while the mice were awake, cerebrovascular hemodynamic changes were recorded for 5 min, and images were continuously collected for 95 min following the anesthetic injection. The image acquisition interval was set to 30 s.

## Results

### Mouse Brain Metabolome Atlas

**Fig. 1:**
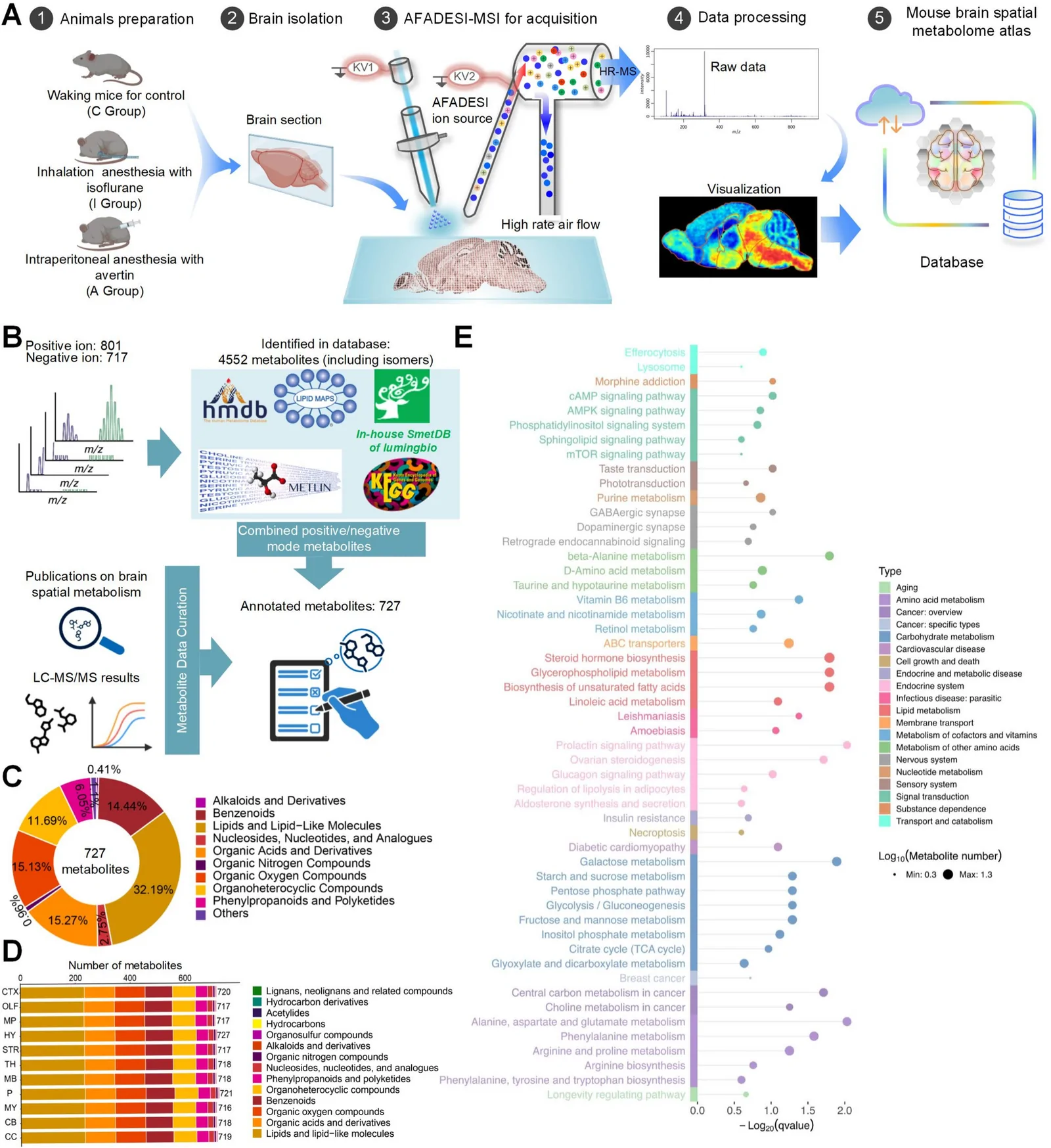
Overview of mouse brain metabolome mapping. **A** The workflow diagram for acquiring spatial metabolomics data from awake and anesthetized mouse brains. **B** Strategy for metabolite annotation. **C** The chemical compositions of the brain metabolome in awake mice were obtained through mass spectrometry imaging (MSI), and classified by ClassyFire to categorize all annotated metabolites. **D** The number of metabolites in various brain regions. **E** The number of metabolites in the brain within KEGG-based pathway sets. (The *q*-value represents the FDR correction applied to the *P*-value in the hypergeometric test).

To investigate the effects of anesthesia on brain metabolism and to provide researchers with reference information on the spatial distribution of brain metabolism in anesthetized mice, we studied nine male wild-type mice, which were divided into three groups: awake (control group), isoflurane-based inhalation anesthesia (isoflurane group), and avertin-based intraperitoneal anesthesia (avertin group). After euthanasia by cervical dislocation, brain samples were rapidly collected, and the preprocessed brain tissues were sectioned into 10 μm successive sagittal slices. The data were acquired using the AFADESI-MSI platform in both positive and negative ion modes. Brain regions were segmented based on the image features of adjacent brain slices stained with hematoxylin and eosin, with reference to the Allen Mouse Brain Atlas. The regions included in this study were the cerebral cortex (CTX), corpus callosum (CC), hippocampus (HP), thalamus (TH), hypothalamus (HY), cerebral nuclei (CNU), olfactory bulb (OLF), pons (P), cerebellum (CB), midbrain (MB), and medulla (MY). Additionally, we created a freely accessible database for interactive exploration of the brain metabolome atlas available at https://mbsma.liulab.cloud/#/dashboard. Fig. 1A illustrates the study design.

Based on the AFADESI-MSI assay, 1,395 ions were detected in the negative ion mode, with annotation information available for 717 ions. In the positive ion mode, 1,220 ions were detected, with annotation information for 801 ions (metabolite annotations for both positive and negative ion modes are provided in Supplementary Data 2). Simultaneously, we conducted a routine metabolic analysis of the residual brain tissue using the LC-MS/MS assay (Fig. S1). In the negative ion mode, 1,734 ions were detected, with annotations for 1,102 ions. In the positive ion mode, 3,716 ions were detected, with annotations for 2,462 ions (annotated metabolite matrix: Supplementary Data 3).

To improve annotation confidence, we first merged the metabolites detected by AFADESI-MSI in the positive and negative ion modes, as shown in Fig. 1B. We then annotated metabolites based on multiple databases, including HMDB, LipidMaps (V2.3), METLIN, the in-house SmetDB database, and KEGG. Metabolites that were consistently annotated in both LC-MS/MS and AFADESI-MSI analyses were retained, as were those reported in previous studies of brain spatial metabolomics [24, 29]. In total, 727 metabolites were identified. A list of manually curated annotations is provided in Supplementary Data 4. As shown in Fig. 1C, the proportions of metabolite species detected by spatial metabolomics were highly comparable to those detected by conventional metabolic analysis, indicating high consistency between the qualitative results obtained from the two techniques.

Owing to the high endogenous lipid content in the brain, lipids and lipid-like molecules accounted for the largest proportion of identified metabolites in the brain metabolome (Fig. 1C). Additionally, the brain contained the following types of compounds: organic acids and their derivatives, organic heterocyclic compounds, organic oxygen compounds, benzenoids, phenylpropanoids and polyketides, nucleosides, nucleotides and analogues, organic nitrogen compounds, organosulfur compounds, and others. As shown in Fig. 1D, the vast majority of brain metabolites were distributed across 11 brain regions with a similar distribution of metabolite types. The pathway enrichment results of these brain metabolites are shown in Fig. 1E, where a large number of metabolites were associated with metabolic pathways, such as carbohydrate metabolism, lipid metabolism, amino acid metabolism, and endocrine systems.

### Anesthesia Impact on the Brain Spatial Metabolome

**Fig. 2:**
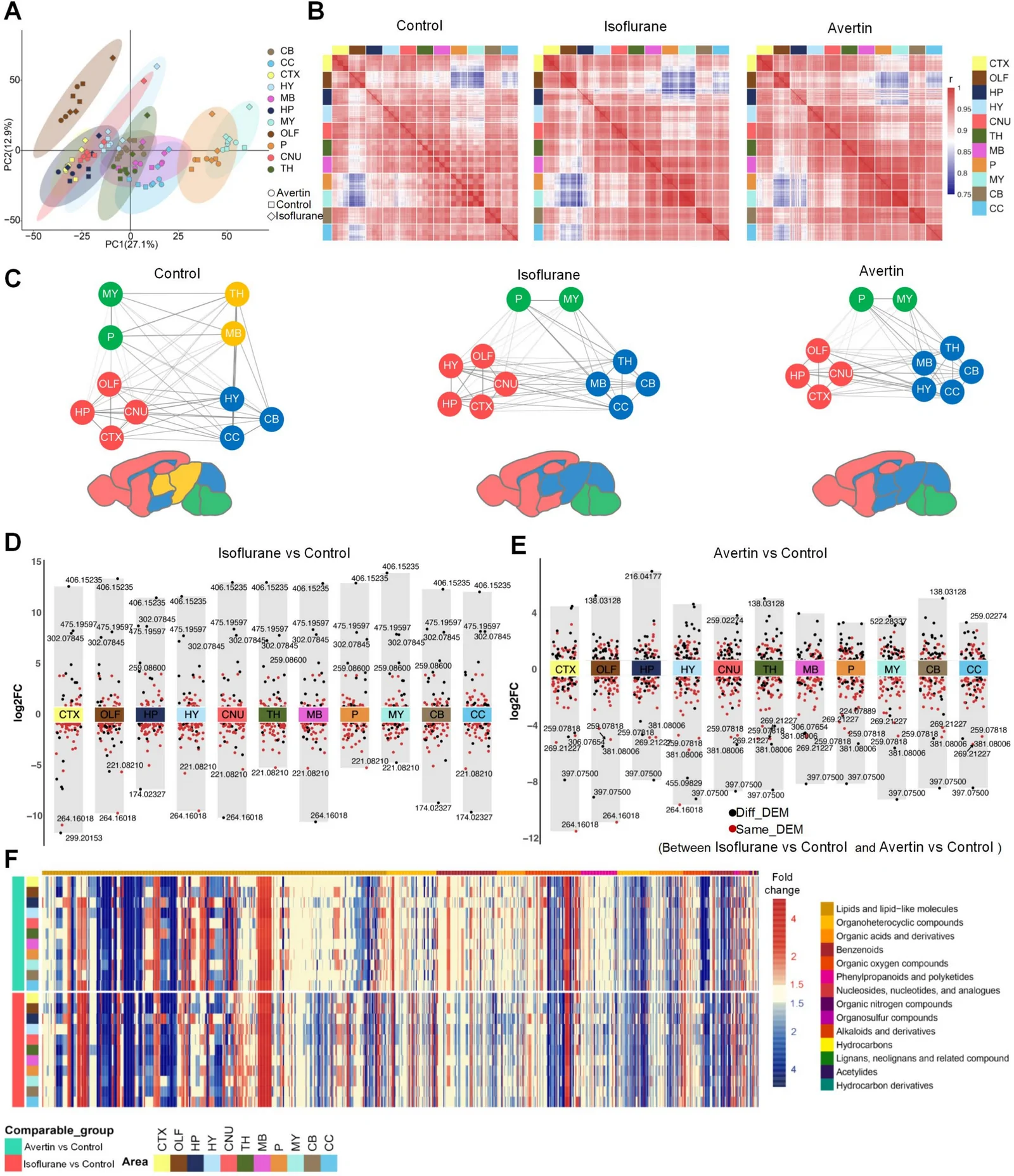
Impact of anesthesia on the mouse brain spatial metabolome. **A** Principal component analysis (PCA) of brain regional metabolomic profiles under different anesthesia conditions. **B** Heatmap matrix shows pairwise Spearman correlations between brain regions in awake and anesthetized mice. **C** Network diagrams are constructed based on the correlation analysis results within brain regions. (The thickness of the lines corresponds to the magnitude of the correlation coefficient: the thicker the line, the stronger the correlation coefficient between nodes, indicating higher interregional brain connectivity.) Brain regions with the same color represent highly correlated modules. **D**, **E** Volcano plot of differential metabolites across brain regions under different anesthesia conditions (each point represents the differential ions within the corresponding group). The top 5 based on the absolute value of log_2_FC are labeled with *m/z*. (log_2_FC > 1.5 and log_2_FC < −1.5). **F** Heatmap of metabolites with >1.5-fold changes between anesthesia groups. Metabolites are categorized by ClassyFire.

When the PCA sample plots were colored according to different parameters, such as brain region, clear biological differences were apparent (Fig. 2A). Fig. 2A illustrates the metabolic phenotypes of different brain regions represented by distinct colors. Sample points from the same brain region were clustered in proximity, indicating a high degree of similarity in primary metabolic features within the same region. The occurrence of relatively dispersed points within a single brain region may be attributed to the effects of anesthesia. Notably, sample clusters from brain regions such as CTX, TH, CNU, HY, and HP were positioned near each other and were clearly separated from regions in the brainstem such as P, MY, and OLF. This observation aligns with the biochemical distinctions among the brain regions previously reported in similar studies [29, 43]. Thus, metabolic phenotypes adhere to the major structural subdivisions of classical brain anatomy. TH, located between the cerebrum and brainstem clusters, showed the smallest number of metabolites that were significantly over- or under-expressed compared to other regions (Fig. S2**)**. This suggests that the TH region is highly interconnected with both the cerebrum and brainstem regions, serving as a hub for transmitting incoming information to adjacent brain regions [44].

Next, we explored the correlations among brain metabolites to investigate specific metabolic differences across various brain regions under different anesthetic conditions. A detailed analysis of the Spearman rank correlation matrices for all 11 brain regions (Fig. 2B) revealed that the distribution patterns of the correlation heat maps across the brain regions were consistent with the findings of other studies [18, 29]. Within the structural subdivisions of the brain, metabolomics showed highly positive correlations except in the brainstem regions (MY and P). Furthermore, Fig. S2 indicates that the spatial metabolomic differences across the brain regions were primarily attributed to lipids and lipid-like molecules. Specifically, lipid metabolites were less abundant in the cerebrum (CTX, OLF, HP, HY, and CNU) but more abundant in the brainstem (P and MY). This was consistent with the results of previous studies [45, 46]. These metabolomic differences between the cerebrum and the brainstem reflect distinct functional specializations.

Networks were constructed using Pearson’s coefficients and significant correlations (*P* < 0.05), resulting in four modules for awake mice and three modules for mice under anesthesia with isoflurane and avertin (Fig. 2C). Notably, the TH and MB brain regions formed independent modules only in the awake group, whereas in the anesthesia groups, they were integrated into modules with other brain regions. These findings indicate substantial metabolic changes in the thalamus and midbrain between the awake and anesthetized states. Previous studies have shown that nuclei or brain regions, such as the thalamus, midbrain, and hypothalamus, play critical roles in the loss and recovery of consciousness induced by general anesthetics and sleep. Modulation of the excitability of these key nuclei can influence anesthesia or sleep states [47]. Recent findings on the differential activation of brain regions by the anesthetics ketamine and isoflurane indicate that isoflurane predominantly stimulates subcortical regions, particularly the hypothalamus, aligning with the bottom-up mechanism of anesthesia-induced loss of consciousness [48]. Our metabolomics-based network analysis further supports distinctions in the role of the HY region between isoflurane and avertin anesthesia: (1) for inhalation anesthesia with isoflurane, the HY region clustered with OLF, HP, CTX, and CNU within the same module; (2) for intraperitoneal anesthesia with avertin, the HY region formed a module with TH, CB, CC, and MB. These differences are likely related to the bottom-up mechanism and top-down mechanism underlying anesthesia-induced loss of consciousness [49].

Further comparative analyses of differential metabolites (DEMs) across different brain regions under various anesthesia protocols were conducted. As shown in Fig. 2D and E, the number of DEMs in each brain region under isoflurane anesthesia was significantly lower than that under avertin-based anesthesia. Moreover, the top five DEMs under isoflurane anesthesia, based on the absolute value of log_2_FC, were predominantly upregulated, whereas the DEMs under avertin anesthesia were mainly downregulated. Additionally, both anesthesia protocols shared a considerable number of common DEMs.

In addition, we observed that lipids and lipid-like molecules accounted for a large proportion of the DEMs under different anesthesia methods (Fig. 2F); among these, glycerol 3-phosphate (G3P) is an important synthetic component in energy metabolism/storage (glycerol lipids) and is also a constituent of lipid rafts in cell membranes. Previous reports have indicated that inhalational anesthetics disrupt lipid rafts on cell membranes, which are key factors in cellular signaling processes [50]. The results in Fig. S3A confirm previous findings, showing a significant increase in G3P levels in cortical regions and other brain areas under isoflurane anesthesia compared to the awake group [51]. Our results provide direct evidence supporting the impact of anesthetics on lipid metabolism [52]. Fig. S3B indicates that the nucleoside metabolite uridine is significantly downregulated in the anesthetized state compared to the awake state and is notably lower under isoflurane anesthesia than under avertin anesthesia. This is consistent with the results of previous studies showing that uridine levels are upregulated during wakefulness [53, 54].

**Fig. 3:**
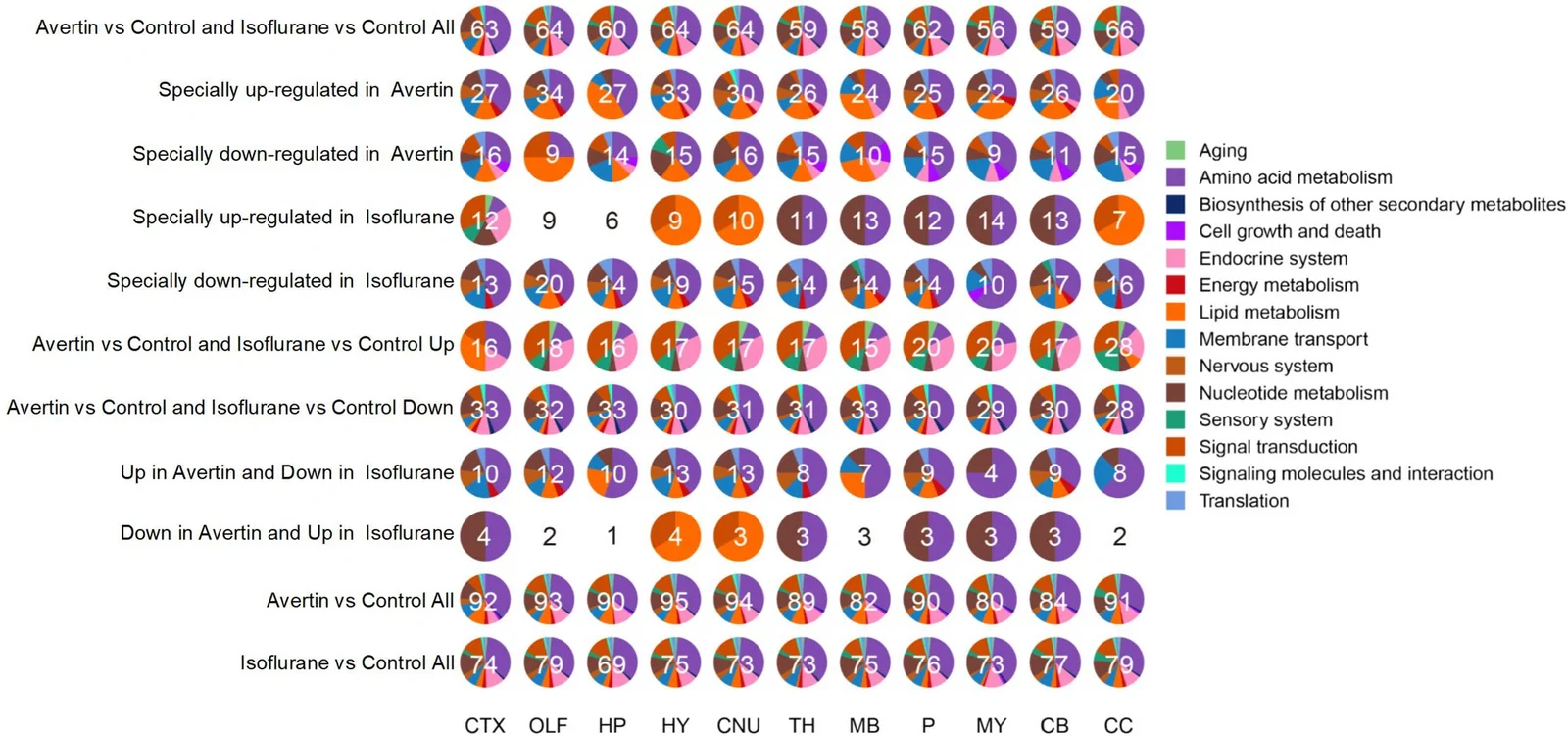
The number of differential metabolites (DEMs) across brain regions in different anesthesia comparison groups and their proportions in the associated metabolic pathways. DEMs were categorized based on their regulation patterns in Avertin vs. control and isoflurane vs. control comparisons, including shared DEMs, anesthesia-specific up- or down-regulated DEMs, concordantly regulated DEMs in both comparisons, and oppositely regulated DEMs between Avertin and Isoflurane. Categories also include all DEMs identified in each comparison. Numbers indicate the counts of DEMs.

Fig. 3 shows the number of DEMs in each brain region across different anesthetic comparison groups, as well as their enrichment in various metabolic pathways. The counts of DEMs in the avertin group were significantly higher than those in the isoflurane group, with the majority of common DEMs being involved in amino acid metabolism and nervous system pathways. The counts of DEMs upregulated only in the avertin group were notably higher than those of downregulated DEMs. In contrast, the number of DEMs that were upregulated only in the isoflurane group was lower than that in the downregulated group. A large proportion of the DEMs upregulated exclusively in the avertin group were involved in amino acid metabolism. In the isoflurane group, a large proportion of exclusively downregulated DEMs were also involved in amino acid metabolism. The number of upregulated DEMs common to both the isoflurane and avertin groups was clearly lower than that of the downregulated DEMs, with the upregulated common DEMs primarily involved in the endocrine and nervous systems, and the downregulated common DEMs mainly involved in amino acid metabolism. Moreover, the number of shared DEMs that were upregulated in the avertin group and downregulated in the isoflurane group was significantly greater than the number of shared DEMs that were downregulated in the avertin group and upregulated in the isoflurane group. Additionally, the proportions of these specific metabolic pathways varied considerably across different brain regions.

### The Effect of Anesthesia on Brain Neuroactivity-Related Metabolites

**Fig. 4:**
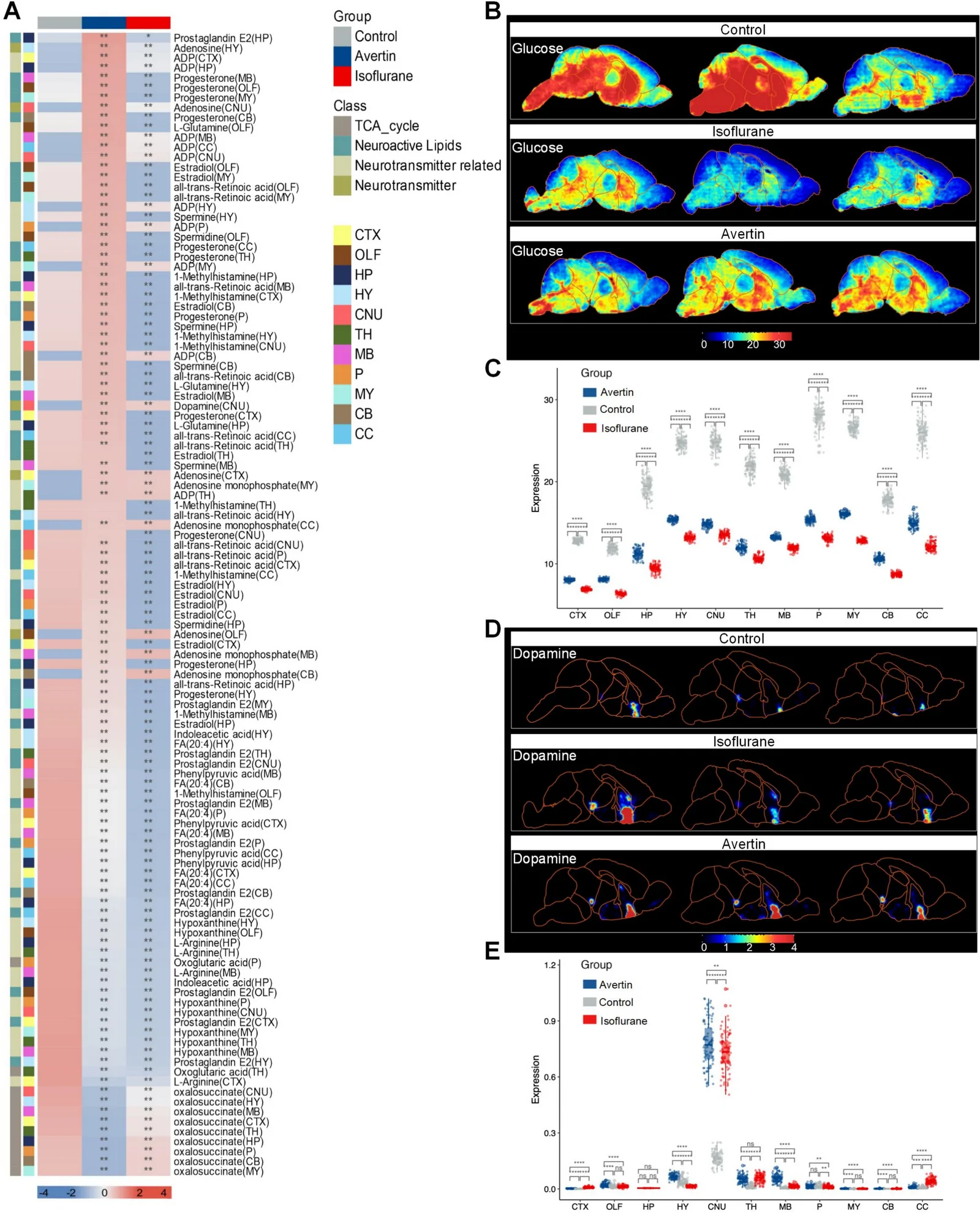
Anesthesia alters the expression of neuroactivity-associated metabolites. **A** Expression levels of metabolites associated with the tricarboxylic acid cycle (TCA cycle), neutral lipids, neurotransmitters, and neurotransmitter-related pathways across different anesthesia groups. The heatmap colors represent metabolite abundance (*t*-test, compared to the mean expression levels in the control group; *** indicates *P* < 0.001, ** indicates 0.001 ≤ *P* < 0.01, and * indicates *P* ≤ 0.05). **B–E** The glucose (*m/z* 215.03273) (**B**) and dopamine (*m/z* 154.08596) (**D**) distribution of the brain in different anesthesia groups (*n* = 3 mice per group). Statistical analysis of glucose (**C**) and dopamine (**E**) expression across different brain regions under various anesthetic conditions. (Each point represents the mean value obtained by repeated resampling of all pixels within the same group; 100 data points were generated for each group) (*t*-test, **** indicates *P* < 0.0001, *** indicates 0.0001 ≤ *P* < 0.001, ** indicates 0.001 ≤ *P* < 0.01, and * indicates 0.01 ≤ *P* ≤ 0.05; NS, *P* > 0.05) The metabolites can be queried online in the database (https://mbsma.liulab.cloud/#/dashboard).

General anesthesia typically reduces oxygen and energy consumption and inhibits metabolic activity in the brain and other organs, which may be caused by the low efficiency of the tricarboxylic acid cycle (TCA cycle) through modulation of mitochondrial function. A previous study showed that almost all general anesthetics exhibit inhibitory effects on mitochondrial function [55]. Consequently, the generation or utilization of TCA cycle intermediates (such as oxalosuccinate) may decrease with metabolic suppression induced by anesthesia, which is consistent with our finding that oxalosuccinate and its isomers were significantly reduced in the avertin-anesthetized group (Fig. 4A**)**. Moreover, glucose, one of the primary fuel sources for the TCA cycle, provides key intermediates and reducing equivalents necessary for the operation of the cycle and is also the main metabolic fuel for the brain under basal aerobic conditions [56]. In line with previous studies [57, 58], we found that anesthesia caused a significant reduction in glucose levels across all brain regions (Fig. 4B, C), which were lower in the isoflurane group than in the avertin group.

Furthermore, we found that anesthesia also affected the expression levels of neuroactive lipids, including prostaglandin E2 (PGE2), progesterone, β-estradiol, and all-trans-retinoic acid (Fig. 4A and Fig. S4). Compared with the awake state, the avertin group showed elevated levels of these neural lipids in some brain regions, whereas the isoflurane group showed significantly reduced levels. PGE2 is involved in the regulation of synaptic activity and plasticity [59]. Fig. S4 shows that PGE2 significantly decreased after anesthesia compared with the awake state, supporting the notion that endogenous PGE2 in the brain plays a role in regulating the physiological mechanisms underlying sleep-wake activities [60]. Progesterone and β-estradiol are important sex hormones, with β-estradiol having neurotrophic and neuroprotective effects [61]. Progesterone receptors are primarily concentrated in the brain and play a significant supportive role in the myelin sheath surrounding neurons. Moreover, progesterone performs various non-reproductive functions in the central nervous system, including the regulation of cognition, mood, inflammation, mitochondrial function, neurogenesis and regeneration, myelination, and recovery after traumatic brain injury [62]. Retinoic acid plays a critical role in the development, regeneration, and maintenance of the nervous system [63] and is also involved in the regulation of sleep [64], with levels decreasing significantly in the isoflurane group. Our results indicate that anesthesia, especially inhalational anesthetics such as isoflurane, can influence the levels of sex hormones in the brain.

Further analysis was conducted on changes in the abundance of neurotransmitters and their associated metabolites under different anesthetic conditions. Among these, dopamine promotes wakefulness under both anesthesia [65] and sleep [66]. The brain metabolome atlas showed high enrichment of dopamine and its metabolites in CNU, consistent with previous studies on the regional distribution of dopamine in the brain [29]. Under anesthetic conditions, the abundance of dopamine was significantly higher than that in the awake state (Fig. 4D, E). Previous reports have indicated that isoflurane can potentiate the ability of dopamine uptake inhibitors to increase brain interstitial dopamine levels [67], which is consistent with our results. Additionally, adenosine, a molecule that promotes sleep, can inhibit cellular excitability, and many types of general anesthetics lead to an increase in extracellular adenosine levels [68]. Similarly, as shown in Fig. S5, we found that adenosine levels in various brain regions under anesthesia were higher than those in the awake state, which aligns with current research findings [34].

### Mouse Brain Spatial Metabolome Database

**Fig. 5:**
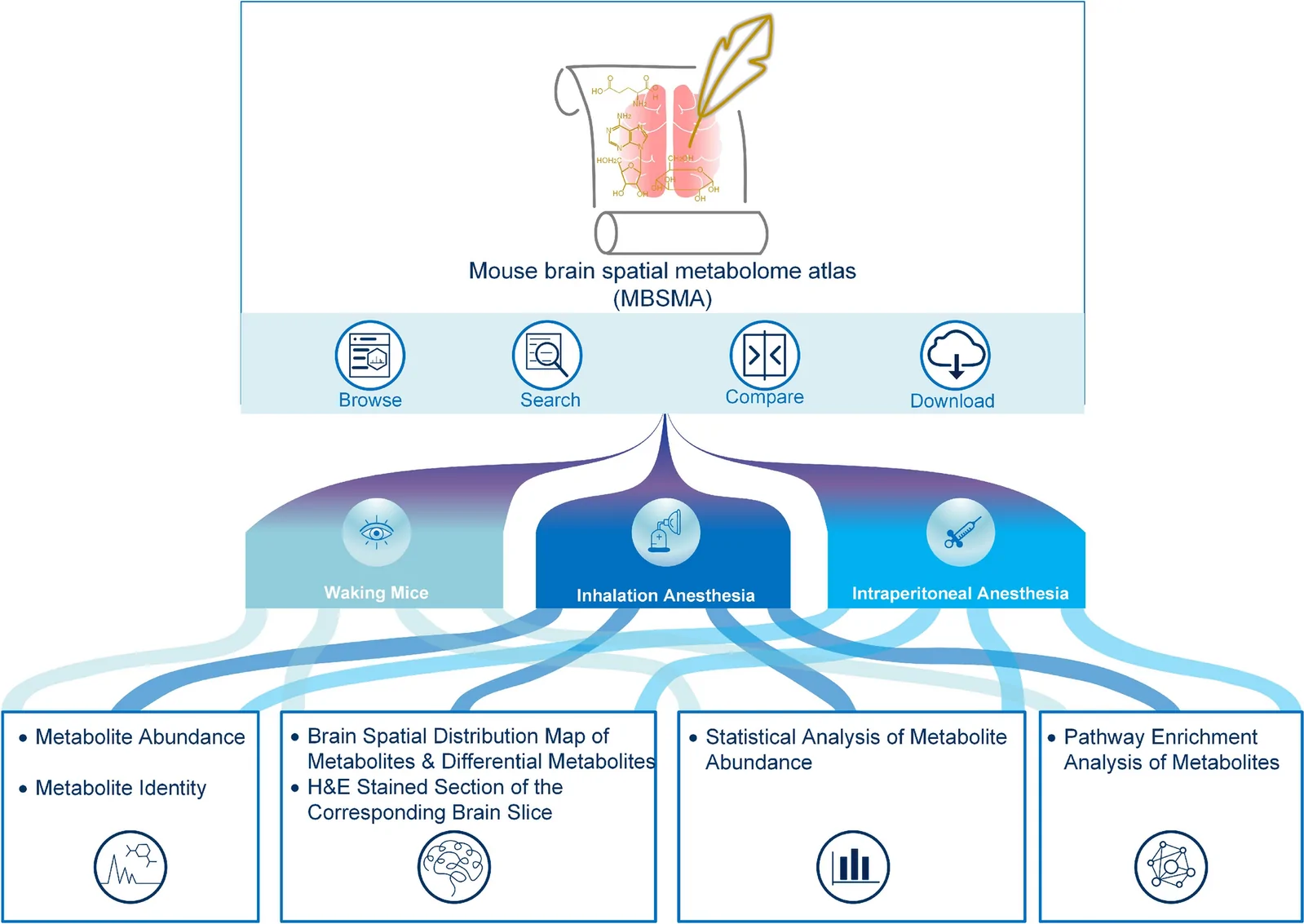
Schematic overview of the MBSMA. The database integrates spatial metabolomic data from mouse brains under diverse experimental conditions, including waking, inhalation anesthesia, and intraperitoneal anesthesia. Core functionalities enable users to browse, search, compare, and download datasets. These modules provide metabolite abundance profiling, brain spatial distribution mapping, statistical analysis, pathway enrichment, and identification of DEMs.

Alterations in metabolite profiles during anesthesia can significantly affect postoperative recovery, tissue oxidative stress, and infection risk. Moreover, anesthesia-induced metabolic changes in mice may have affected the interpretation of the experimental results. To facilitate the convenient retrieval and analysis of spatial brain metabolome data under both awake and anesthetized conditions, we developed a user-friendly online database (https://mbsma.liulab.cloud/#/dashboard). The Mouse Brain Spatial Metabolome Atlas (MBSMA) enables users to browse, search, and compare metabolite information across different brain regions under various anesthetic methods (inhalation anesthesia and intraperitoneal anesthesia) and download the corresponding images and relevant data tables (as shown in Fig. 5). The platform provides detailed data on metabolite abundance, identity, spatial distribution, H&E-stained brain sections, and MSI images as well as statistical analyses of metabolite levels and pathway enrichment. Similar datasets, such as age-related brain metabolic profiles and brain genomic atlases, serve as valuable tools for neuroscience research [20, 23, 29]. Collectively, this resource facilitates comprehensive exploration of spatially resolved metabolic dynamics in neurobiological studies.

### Alterations in Cerebral Microvascular Blood Flow and Oxygen Levels under Different Anesthesia Methods

**Fig. 6:**
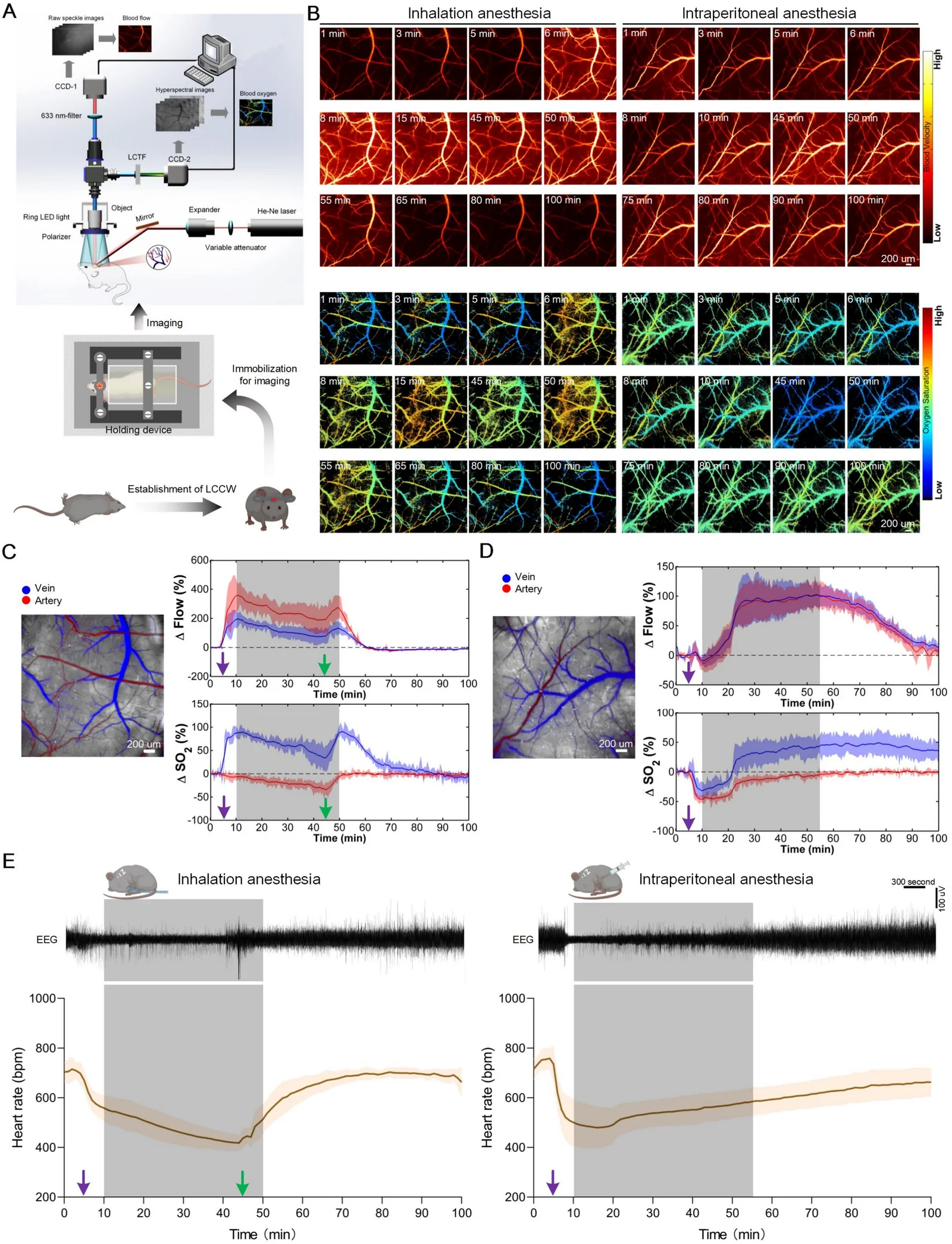
The effects of anesthesia on cortical vascular hemodynamics. **A** Schematic of the experimental preparation and imaging system for high-resolution *in vivo* monitoring of cortical microvascular blood flow and blood oxygen. **B** Time series maps showing the changes in cortical vascular blood flow and blood oxygen at different time points of the entire process from wakefulness to anesthesia and recovery under different anesthetic conditions (inhalation anesthesia with isoflurane and intraperitoneal anesthesia with avertin). Scale bar, 200 μm. **C**, **D** Analysis of blood flow and oxygen saturation (SO_2_) changes in brain arteriovenous microvasculature during inhalation anesthesia (**C**) and intraperitoneal anesthesia (**D**), with segmentation of cortical arteries and veins (*n* = 6, mean ± standard deviation). Scale bar, 200 μm. **E** Representative electroencephalography (EEG) traces and quantified heart rate dynamics in mice under inhalation versus intraperitoneal anesthesia over time (*n* = 5, mean ± standard deviation). The shading in the statistical plot represents the phase of no response to toe pinching with forceps following anesthesia. The purple arrows represent the time point when anesthetic drugs are administered, and the green arrows indicate the time point when isoflurane inhalation is terminated.

Using the LCCW combined with LSCI/HSI imaging techniques, we non-invasively monitored cortical vascular blood flow and blood oxygen levels with high resolution. We dynamically monitored the changes in cerebrovascular function in mice during the entire process, from wakefulness to anesthesia and back to recovery (Fig. 6A). Fig. 6B shows the blood flow and oxygen saturation (SO_2_) maps of the cortical vessels under different anesthesia methods. The results showed that the two anesthesia methods had distinct effects on neural activity and energy metabolism, which was consistent with the results of the metabolic analysis presented above. These findings suggest an association between metabolic alterations and cerebrovascular dynamics.

Furthermore, by leveraging the differences in extinction coefficients between oxy- and deoxyhemoglobin, the arteries and veins were identified and color-coded in red and blue, respectively. Statistical analyses were conducted to evaluate the changes in blood flow and blood oxygen levels in the arterioles and venules (Fig. 6C and D). The results revealed that under isoflurane anesthesia, vascular blood flow increased rapidly following anesthesia induction before gradually declining. Notably, the fluctuations in SO₂ levels differed significantly between the arteries and veins. Upon cessation of isoflurane inhalation, blood flow quickly returned to baseline levels, with arterial SO₂ recovering to baseline more rapidly than venous SO₂. In contrast, under avertin anesthesia, the vascular blood flow increased gradually after induction and then stabilized at a steady level. Compared to isoflurane anesthesia, recovery of blood flow to baseline levels took longer following avertin anesthesia. Venous SO₂, however, remained consistently elevated throughout the observation period. These findings indicate that different anesthetic approaches have differential effects on the regulation of cerebral blood flow, oxygenation, and metabolism, and they contribute to a better understanding of the bidirectional interaction between metabolism and hemodynamics during anesthesia.

Consistent with the observed cerebrovascular dynamics, the electrophysiological and systemic physiological parameters exhibited distinct patterns under different anesthetic conditions. Under inhalation anesthesia, EEG recordings showed gradual suppression of neural activity following induction, accompanied by a pronounced decrease in heart rate (Fig. 6E). Upon cessation of isoflurane inhalation, both EEG activity and heart rate recovered quickly and returned to baseline levels within a short time window. In contrast, intraperitoneal anesthesia induced a rapid suppression of EEG activity, with the heart rate decreasing sharply at induction but recovering slowly and remaining below baseline for an extended period. Notably, the recovery of neural activity and heart rate under intraperitoneal anesthesia was markedly delayed compared with that under inhalation anesthesia, which was consistent with the slower recovery of cerebral blood flow and oxygen dynamics. These findings indicate that intraperitoneal anesthesia induces rapid suppression of neural activity and systemic physiology, whereas inhalation anesthesia enables more controllable and rapidly reversible suppression, highlighting the fundamentally distinct temporal dynamics despite comparable inhibitory depths.

## Discussion

In spatial metabolomics, each pixel of a sample requires mass spectrometry (MS) scanning, and multiple steps such as sample analysis, ion identification, and image generation must be completed within an extremely short timeframe. Tandem mass spectrometry (MS/MS) scans of a large number of ions require further fragmentation of the ions into smaller fragments, followed by detection and analysis *via* separation and screening techniques. This process increases the number of MS cycles per pixel and significantly prolongs the scanning time and energy consumption while reducing key performance parameters such as instrument sensitivity and resolution. Consequently, weaker signals or unidentifiable peaks may occur, compromising the accuracy and reliability of MS data. To ensure experimental efficiency and data accuracy in MS imaging, only a subset of ions is typically selected for MS/MS scans, prioritizing ions with strong signals and stable reactions for detection and analysis. In this study, we performed in situ MS/MS validation of several metabolites resistant to degradation, including guanosine monophosphate, cholesterol sulfate, 1-methylhistamine, carnosine, phosphatidylcholine [PC(40:6)] and phosphatidylserine [PS(38:6)]. Fig. S6 presents the in situ MS/MS characteristic fragment imaging of these metabolites, along with mirror plots showing the alignment of the in situ MS/MS fragments with those in the standard reference database. These metabolites exhibited similar spatial distribution patterns within the brain regions, and the secondary fragments of the target ions detected in situ aligned closely with those in the standard database. This alignment provides evidence supporting the accuracy of metabolite detection in spatial metabolomics to a certain extent. Furthermore, although the brain sections were obtained from highly similar anatomical locations, only one representative section per mouse was imaged to expedite data acquisition. Future studies should employ serial sectioning to enhance data comprehensiveness.

Additionally, the difference between the changes in glucose (Fig. 4B, C) and the changes in cerebral blood flow and oxygenation (Fig. 6) suggests that the reduction in glucose during anesthesia does not result from a decrease in glucose delivery by the vascular system [57]. Previous studies have indicated that adenosine can regulate blood flow by promoting vasodilation, particularly under hypoxic conditions, where it increases local blood flow to improve oxygen supply [69]. Therefore, elevated levels of adenosine in the brain during anesthesia may have contributed to the increase in cerebral blood flow. The observed differences in the levels of associated metabolites under various anesthesia modalities are likely to be closely linked to changes in cerebral blood flow and blood oxygen levels during the anesthetic process.

Brain tissue was collected using euthanasia procedures that complied with established animal welfare and ethical guidelines. Previous studies have reported that acute euthanasia methods, such as cervical dislocation or decapitation, can induce rapid increases in dopamine levels due to stress-associated neurotransmitter release [20, 70]. Although all animals in this study were subjected to the same terminal procedure prior to tissue collection, dopamine levels were markedly higher in anesthetized animals than in the awake group. If postmortem or agonal effects were the predominant drivers of these changes, a similar direction and magnitude of alteration would be expected across all groups. However, substantially elevated dopamine levels under anesthesia suggest that anesthetic-specific modulation of dopamine may play a more prominent role than terminal stress responses alone. This interpretation is consistent with a previous report showing that certain anesthetic agents can influence dopamine metabolism [67]. Nevertheless, we cannot fully exclude the contribution of postmortem and agonal factors to metabolic changes in the brain. These findings should be interpreted with caution, particularly regarding labile neurotransmitters.

In conclusion, studying the effects of anesthesia on brain metabolism is of great significance, including for understanding the mechanisms of anesthesia and the physiological mechanisms of sleep and wakefulness [71, 72]. Enhancing the safety and efficacy of clinical anesthesia is also crucial. By understanding the dynamic changes in brain metabolism under anesthesia, we can optimize anesthesia management strategies, reduce intraoperative and postoperative brain dysfunction, and further advance the field of neuroscience.

This study provides a comprehensive spatial characterization of anesthesia-associated metabolic alterations; however, it is primarily descriptive in nature and does not establish direct mechanistic links between specific metabolic pathways and functional outcomes. Nevertheless, the generated spatially resolved dataset offers a valuable framework for hypothesis generation. Building on the spatial metabolomics atlas established in this study, future investigations could systematically dissect how distinct anesthetic modalities reprogram brain metabolism in a region-specific manner. By integrating this resource with targeted perturbation strategies as well as genetic and pharmacological manipulations, it may be possible to identify key metabolites and coordinated metabolic networks that govern anesthesia-associated brain states. Furthermore, coupling these approaches with multimodal readouts may provide mechanistic insights into the contribution of metabolic reconfiguration to neurovascular regulation and functional brain states during anesthesia.

This study has certain limitations. To achieve whole-section spatial acquisition within a reasonable timeframe, the MSI workflow relies primarily on MS1-only data, which inherently limits its ability to distinguish structural isomers. Although this issue was minimized by integrating LC-MS/MS data and cross-referencing metabolites reported in other brain spatial metabolomics studies, isomer ambiguity remains a central technical challenge in current MSI-based metabolomics. Another limitation of this study is the relatively small number of biological replicates, which may reduce the statistical power and limit the ability to capture inter-animal variability, although the coefficients of variation (CV) distributions indicate overall measurement stability across experimental groups (Fig. S7). Moreover, although MSI provides high-dimensional spatial data through extensive pixel-level sampling, these measurements do not substitute for independent biological replication. Therefore, these findings should be interpreted with caution. Further studies with larger sample sizes are necessary to validate the robustness and generalizability of the observed metabolic patterns. Moreover, the brain spatial metabolomic reference dataset generated in this study was derived exclusively from adult male mice subjected to different anesthesia conditions. Future investigations should incorporate animals of varying ages and sexes to improve the generalizability of the dataset. This study focused primarily on anesthetic agents that are commonly used in experimental research. Expanding the dataset to include a wider range of anesthetics, particularly those frequently administered in clinical practice, is expected to provide significant clinical insights and reference values.

Another limitation of this study is the lack of cell-type specificity inherent to MSI. Although MSI enables spatially resolved metabolomic profiling, each pixel represents a composite signal from multiple cell types, including neurons, glial cells, and vascular elements. Therefore, the observed metabolic alterations should be interpreted at the regional or tissue level rather than attributed to specific cellular or synaptic processes. Consequently, any inference regarding cell-type-specific metabolic activity remains to be made. Future integration with higher-resolution approaches such as spatial transcriptomics or single-cell omics will be important for resolving the cellular origins of these metabolic patterns.

## Conclusion

In this study, we established a comprehensive spatial metabolomic atlas of the mouse brain under both wakefulness and anesthesia conditions using high-throughput MSI. Our comparative analysis of the effects of various anesthetic modalities on brain metabolism offers new insights into how anesthesia alters metabolic processes in different regions of the brain. Furthermore, the integration of the optical clearing skull window and LSCI/HSI techniques allowed us to dynamically monitor changes in cortical microvascular blood flow and blood oxygen levels, providing additional context for understanding metabolic shifts during anesthesia. The creation of an online spatial metabolome atlas will serve as a valuable resource for the neuroscience and metabolomics communities, facilitating further exploration of brain metabolism and the impact of anesthetics. Overall, our study contributes to a deeper understanding of the complex relationship between brain function, metabolism, and anesthesia.

## Supplementary Information

Below is the link to the electronic supplementary material.

Supplementary file1 (PDF 903 KB)

Supplementary file2 (XLSX 15 KB)

Supplementary file3 (XLSX 1439 KB)

Supplementary file4 (XLSX 2461 KB)

Supplementary file5 (XLSX 216 KB)

## Data Availability

The authors declare that the data supporting the findings of this study are available in the paper and Supplementary Information files. The raw MSI data is available at the platform for metabolite annotation of imaging mass spectrometry data (METASPACE) website (https://metaspace2020.org/api_auth/review prj=32b6f9da-c134-11f0-a049-93deaa829d60&token=ky2iGjgjEAyy). Routine metabolic data from LC-MS/MS can be accessed directly *via* the China National Center for Bioinformation (https://ngdc.cncb.ac.cn/omix; OMIX ID: OMIX009384).
