# Supplementary material for: Mapping Brain Spatial Metabolic Atlas Reveals Anesthesia-Induced Region-Specific Alterations
Source: Neurosci Bull. 2026 Jul 18;42(10):2181–98. doi: 10.1007/s12264-026-01675-6 (PMC13642873; doi:10.1007/s12264-026-01675-6)
Supplement: Supplementary file 1 [file 12264_2026_1675_MOESM1_ESM.pdf]

Supplementary Material

Supplementary Data 1. Model parameters for multivariate statistical analysis.

Supplementary Data 2. List of MSI-based metabolites annotated in both positive and negative ion modes using the Human Metabolome Database (HMDB), LipidMaps (v2.3), METLIN, and the in-house SmetDB database (Lumingbio, Shanghai, China).

Supplementary Data 3. List of metabolites identified by LC-MS/MS analysis.

Supplementary Data 4. Curated list of MSI-based metabolites manually annotated by integrating LC–MS/MS results and metabolite information reported in the literatures.

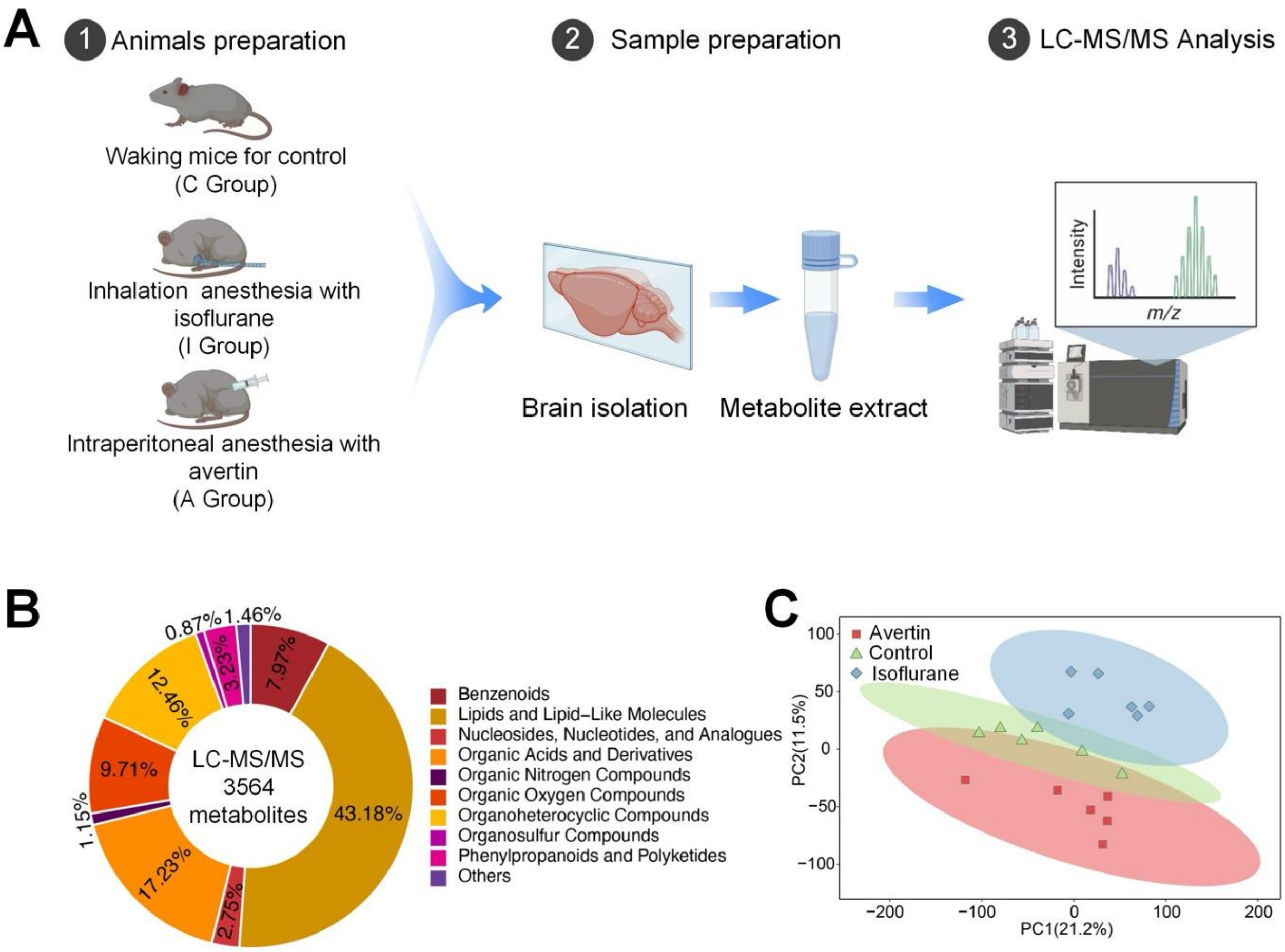

Fig. S1 LC-MS/MS-based metabolomic profiling of mouse brains under different anesthesia conditions. **A** Schematic of the experimental workflow, including animal grouping (awake control, isoflurane anesthesia, and avertin anesthesia). **B** Chemical classification of the 3,564 metabolites identified by LC-MS/MS, grouped according to major metabolite classes. **C** Principal component analysis (PCA) of brain metabolomic profiles from the Avertin, Control, and Isoflurane groups, illustrating anesthesia-dependent metabolic separation.

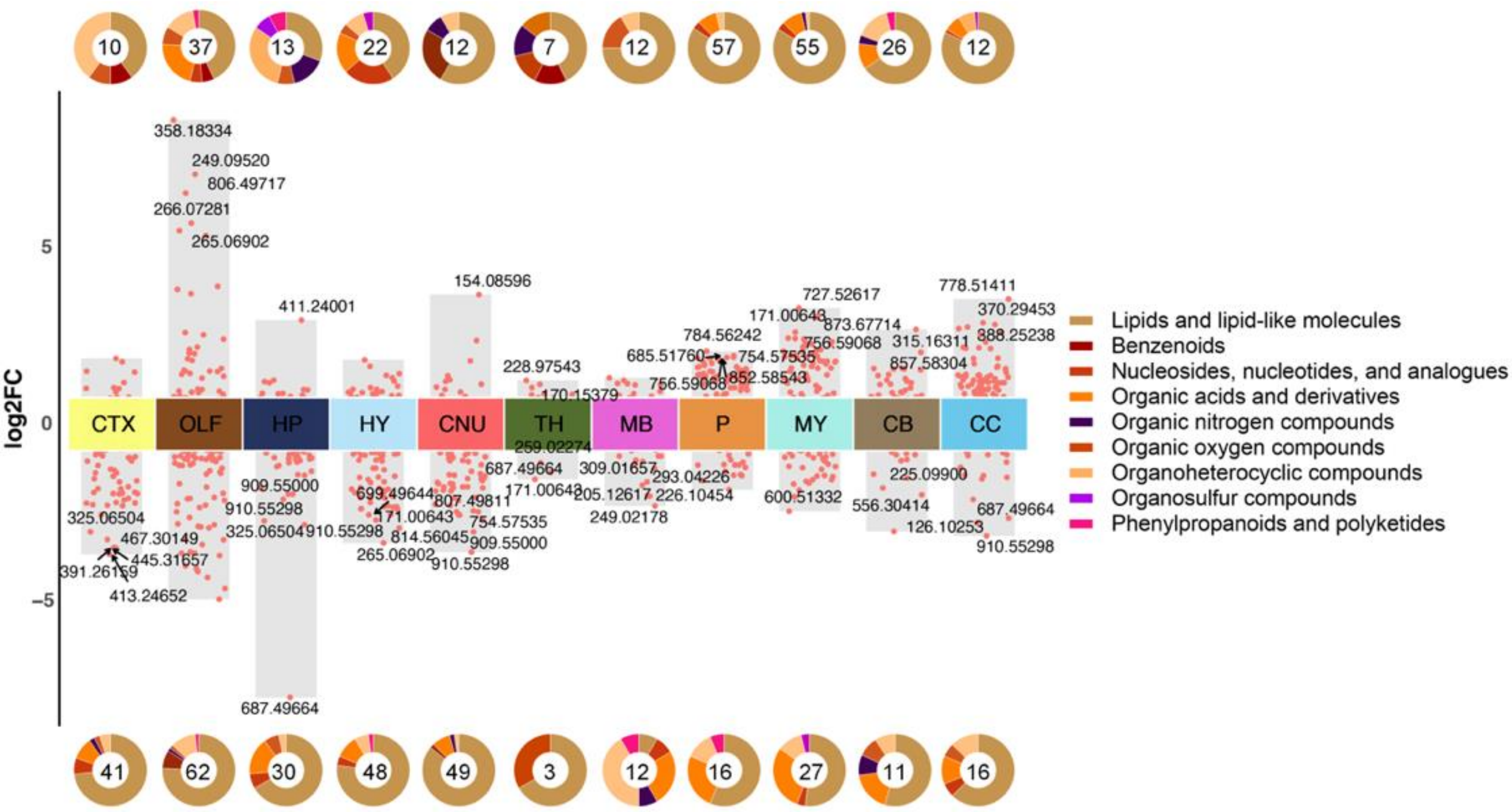

Fig. S2 The spatial metabolomic differences across brain regions. Volcano plot of differential metabolites across brain regions. The circular proportional chart displays the types and quantities of differential metabolites. Cerebral Cortex (CTX), Corpus Callosum (CC), Hippocampus (HP), Thalamus (TH), Hypothalamus (HY), Cerebral Nuclei (CNU), Olfactory Bulb (OLF), Pons (P), Cerebellum (CB), Midbrain (MB) And Medulla (MY)

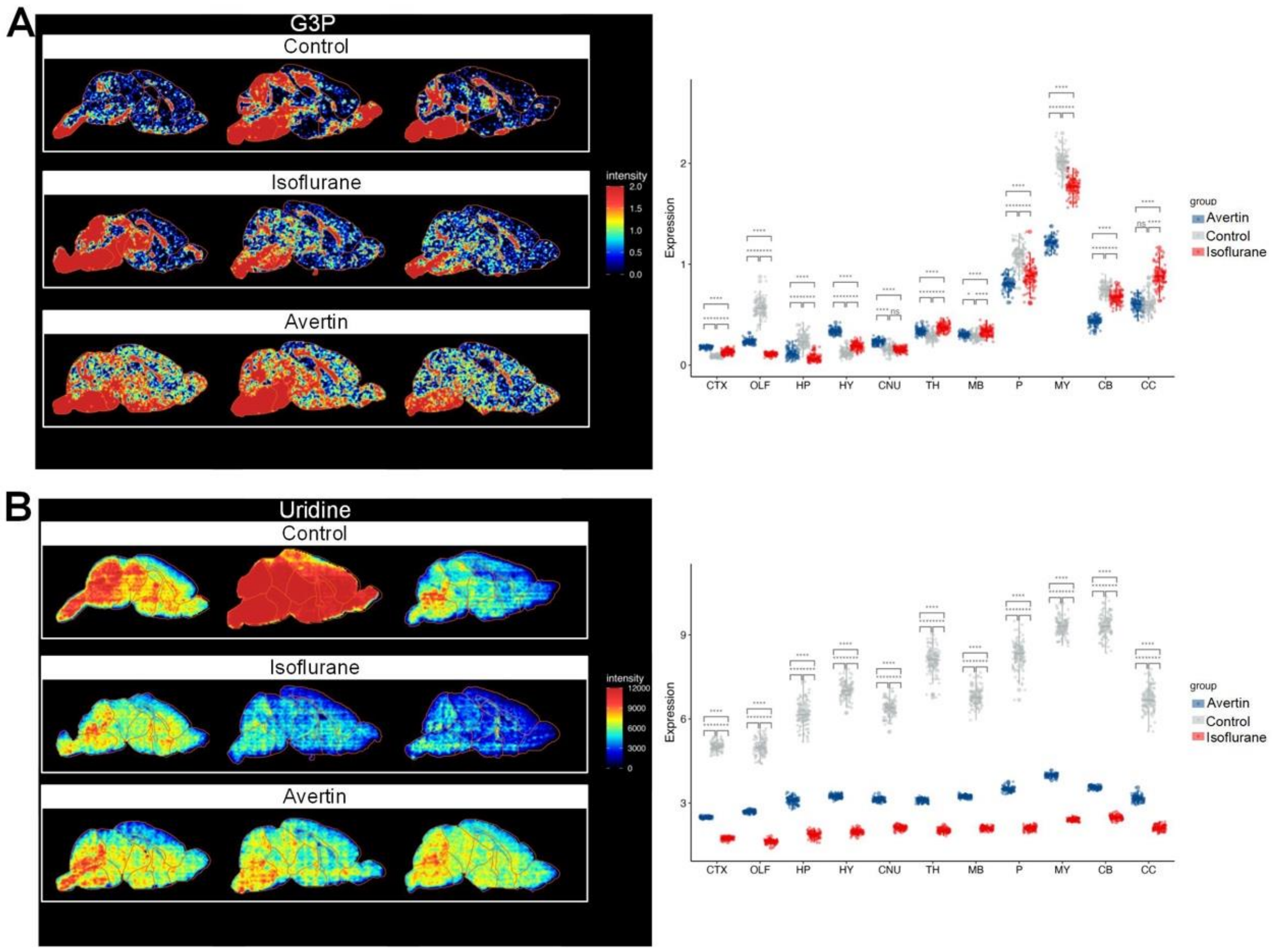

Fig. S3 The glycerol 3-phosphate (G3P) (**A**) and uridine (**B**) distribution of the brain in different anesthesia groups. Statistical analysis of G3P and uridine expression across different brain regions under various anesthetic conditions. (*t*-test, **** indicates $P < 0.0001$, *** indicates $0.0001 \le P < 0.001$, ** indicates $0.001 \le P < 0.01$, and * indicates $0.01 \le P < 0.05$; ns: $P > 0.05$)

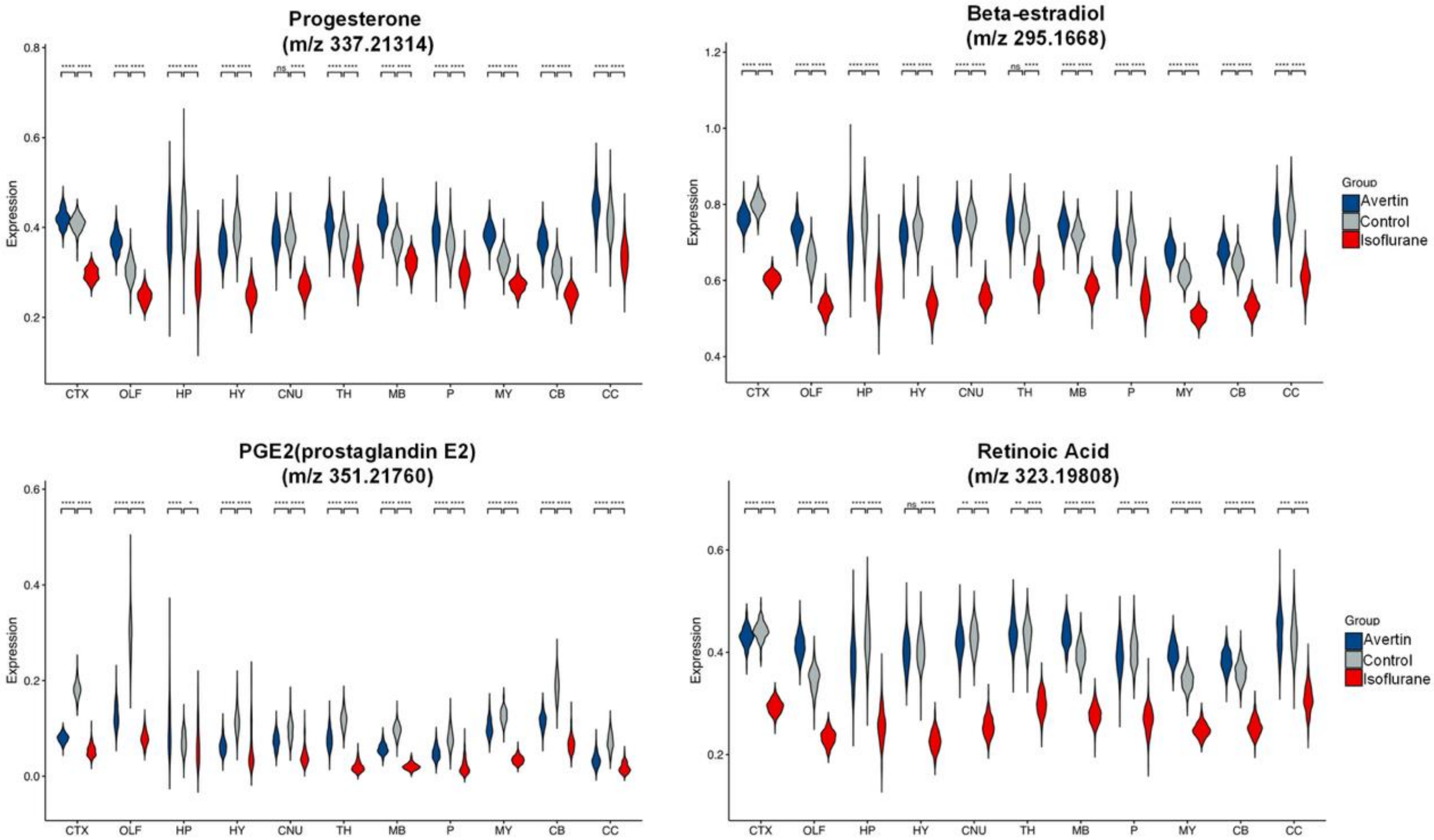

Fig. S4 Statistical analysis of the expression levels of some metabolites (progesterone, β-Estradiol, prostaglandin E2 (PGE2), and retinoic acid) in different brain regions under awake and anesthetized conditions. (*t*-test, **** indicates $P < 0.0001$, *** indicates $0.0001 \le P < 0.001$, ** indicates $0.001 \le P < 0.01$, and * indicates $0.01 \le P < 0.05$; ns: $P > 0.05$)

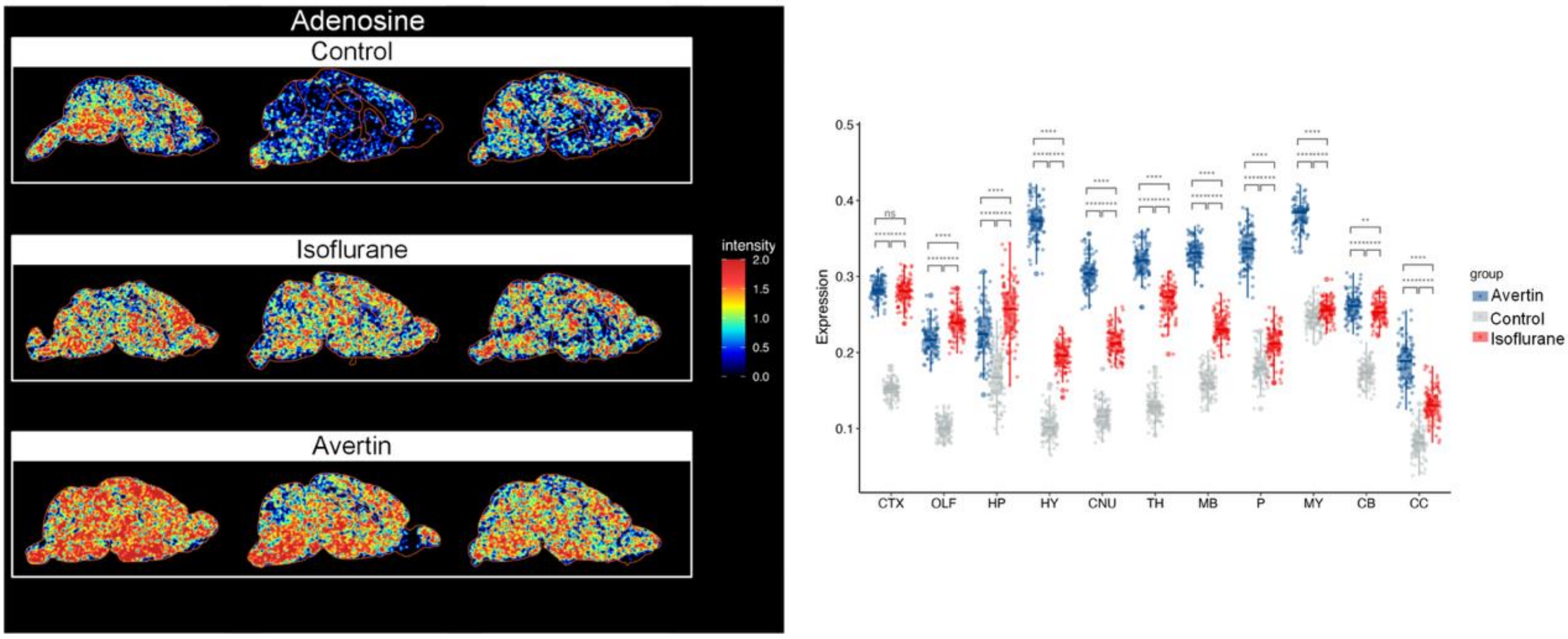

Fig. S5 The adenosine distribution of the brain in different anesthesia groups. Statistical analysis of adenosine expression across different brain regions under various anesthetic conditions. (*t*-test, **** indicates $P < 0.0001$, *** indicates $0.0001 \leq P < 0.001$, ** indicates $0.001 \leq P < 0.01$, and * indicates $0.01 \leq P < 0.05$; ns: $P > 0.05$)

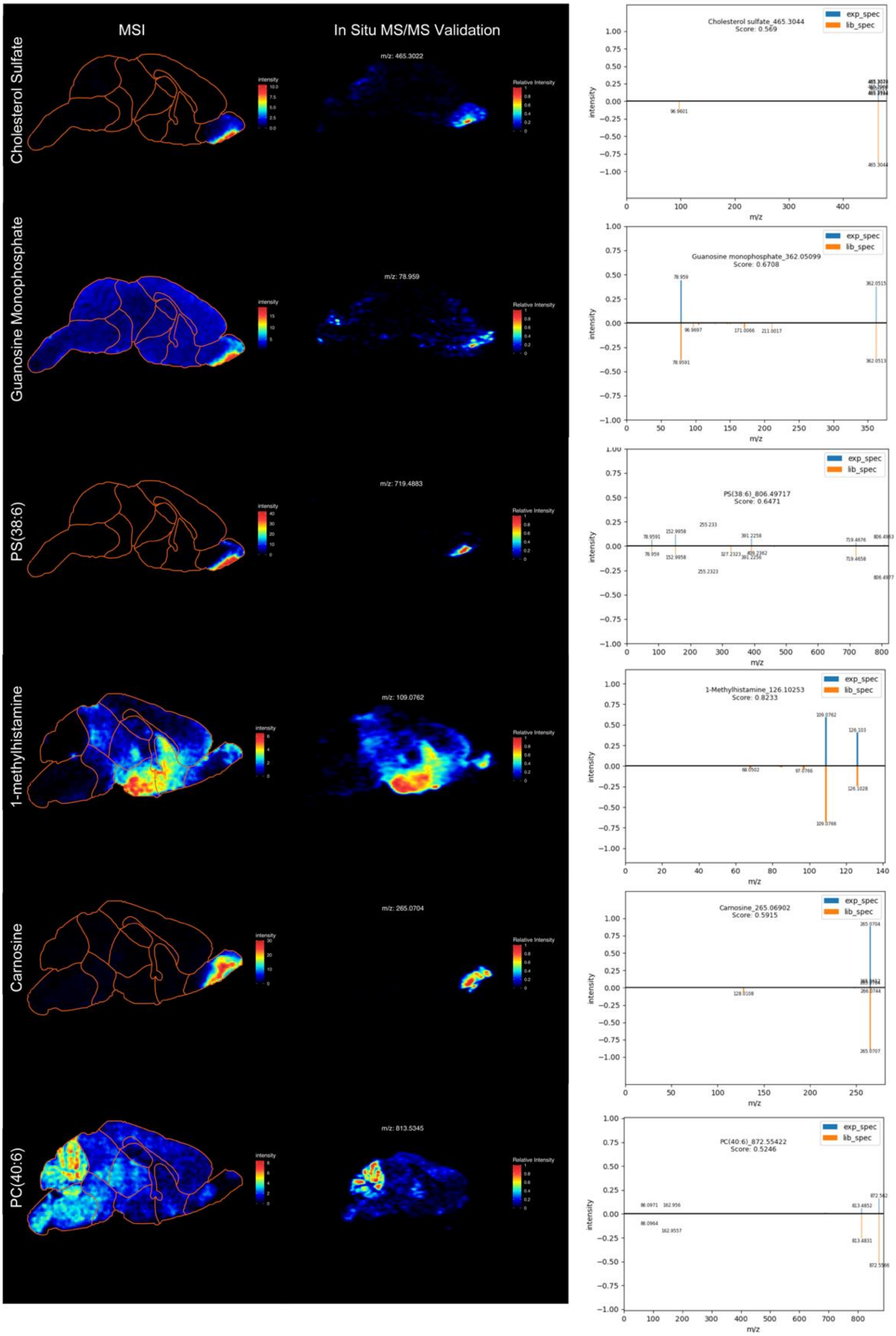
MSI
In Situ MS/MS Validation
Cholesterol Sulfate
m/z: 465.3022
Cholesterol sulfate_465.3044
Score: 0.569
Guanosine Monophosphate
m/z: 78.959
Guanosine monophosphate_362.05099
Score: 0.6708
PS(38:6)
m/z: 719.4883
PS(38:6)_806.49717
Score: 0.6471
1-methylhistamine
m/z: 109.0762
1-Methylhistamine_126.10253
Score: 0.8233
Carnosine
m/z: 265.0704
Carnosine_265.06902
Score: 0.5915
PC(40:6)
m/z: 813.5345
PC(40:6)_872.55422
Score: 0.5246
intensity
Relative Intensity
exp_spec
lib_spec
m/z

Fig. S6 In situ secondary validation of the relatively stable metabolites, including cholesterol sulfate, guanosine monophosphate, phosphatidylserine [PS(38:6)], 1-methylhistamine, carnosine, and phosphatidylcholine [PC(40:6)]. To validate the mass spectrometry imaging (MSI) results, secondary in situ verification was conducted using tandem mass spectrometry (MS/MS) and co-localization analysis. The obtained fragmentation patterns and spatial distributions were consistent with reference spectra and known metabolite localization, confirming the accuracy of the MSI-based annotations.

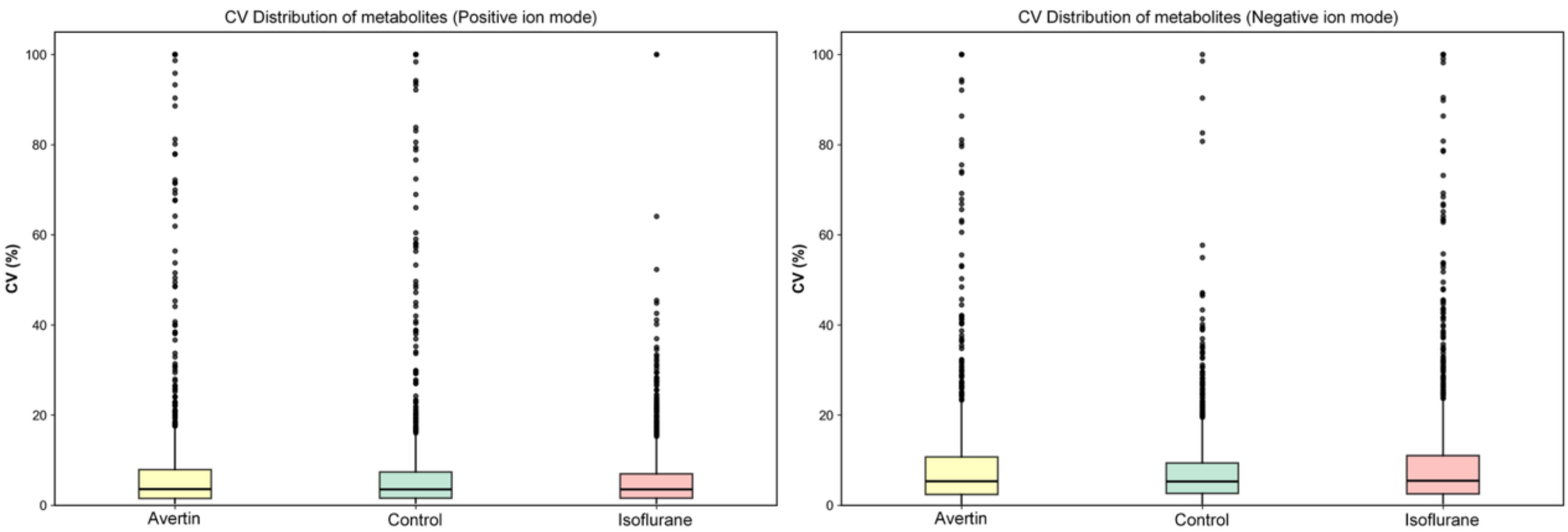

Fig. S7 Coefficient of variation (CV) distributions indicate overall measurement stability across experimental groups. Boxplots depict the distribution of CV values for all detected metabolites in the Avertin, Control, and Isoflurane groups in both positive (left) and negative (right) ion modes. Each dot represents a single metabolite. The majorities of metabolites displayed low CV values (median <10%), suggesting acceptable within-group consistency, although a fraction of metabolites exhibited higher variability.
